# A new *in vitro* rat liver platform capturing biological diversity for drug-induced liver injury assessment and reduction of animal use in drug discovery and development

**DOI:** 10.3389/ftox.2026.1784229

**Published:** 2026-04-28

**Authors:** Pralhad Wangikar, Sara Cherradi, Pradhnya Choudhari, Aditi Wangikar, Vaibhav Madiwal, Salomé Roux, Nisha Banerjee, Pranav Panzade, Pallavi Gangarde, Nithish Kannan, Hong Tuan Duong

**Affiliations:** 1 PRADO Pvt Ltd, Pune, India; 2 PredictCan Biotechnologies, Biopôle Euromédecine, Cap Sigma, Grabels, France

**Keywords:** drug-induced liver injury, individual variability, In vitro-in vivo correlation, reduction of animal use, serum-educated rat liver spheroid model

## Abstract

Drug-induced liver injury (DILI) is a major cause of drug development failure and market withdrawal. Despite the use of animal and human-derived preclinical models, none reliably predict human hepatotoxicity. Conventional animal studies, based on group-level averages, overlook inter-individual variability critical to idiosyncratic DILI. Although several alternatives to animal testing are available, implementation of alternative methodologies into safety evaluations is very slow and to date, no standalone validated alternative models to assess systemic toxicity exist. To address this, we developed a novel serum-educated rat liver spheroid model that captures individual metabolic diversity, allowing DILI to be assessed both *in vitro* and *in vivo* within the same animal. Building on this approach, the present study aimed to validate the model’s ability to detect DILI caused by well-characterized hepatotoxic drugs, including diclofenac and bosentan, in rat models. Rats were treated orally with diclofenac or bosentan for 28 days. Blood was collected pre-dosing to generate individualized spheroids containing rat hepatocytes, stellate cells, and macrophages. These spheroids were exposed to each drug across a ten-point concentration range for 3 days and cell viability was quantified using the CellTiter-Glo ATP assay. Clinical chemistry analyses of ALT, AST, ALP, albumin, and bilirubin were performed at *in vivo* study termination on day 29. *In vitro* data were analyzed using PredictCan-MIND to derive DILI severity scores and correlate *in vitro* cytotoxicity with *in vivo* biomarkers. *In vivo*, diclofenac caused no significant liver enzyme elevations, although some rats, exclusively females, displayed subtle toxicity revealing sex-dependent susceptibility. Corresponding *in vitro* spheroids confirmed hepatocellular injury in these individuals. Bosentan produced a mild cholestatic response *in vivo* without consistent enzyme elevation, while *in vitro* analysis showed clear hepatotoxicity in 8 of 10 rats. Notably, *in vitro* DILI severity correlated strongly with *in vivo* ALP levels, consistent with bosentan’s known cholestatic mechanism. The serum-educated rat liver spheroid model captures inter-individual and sex-related differences in hepatotoxicity and demonstrates translational concordance with *in vivo* cholestatic markers. This approach improves DILI prediction, aligns with the 3Rs principle, and supports a potential 50%–70% reduction in animal use for preclinical liver toxicity testing.

## Introduction

Drug-induced liver injury (DILI) remains one of the most critical obstacles in pharmaceutical development, accounting for nearly 30% of drug development failures and post-marketing withdrawals due to adverse drug reactions (Babai et al., 2021). The unpredictable nature of DILI, particularly its idiosyncratic forms, poses substantial challenges for both patient safety and regulatory approval (European Association for the Study of the Liver, 2019). Despite extensive preclinical testing, hepatotoxic liabilities are frequently identified only in late-stage clinical trials or after market release, underscoring persistent gaps in the translational reliability of current safety assessment strategies (Dirven et al., 2021).

To address this issue, a wide array of preclinical models has been developed, including primary human hepatocytes, liver organoids, and diverse animal models. While these systems have provided valuable mechanistic insights, none has consistently demonstrated robust translatability to human responses (Marshall et al., 2023). In particular, rodent and non-rodent animal models, despite preserving intact immune systems and inter-organ communication, often fail to accurately predict human hepatotoxicity (Roth and Ganey, 2010; Shaw et al., 2010). Moreover, conventional animal studies rely primarily on group-level analyses that compare average responses between treated and control cohorts (van der Goot et al., 2021). Such approaches overlook inter-individual variability, a crucial limitation given that DILI typically affects only a subset of susceptible individuals. In recognition of these shortcomings, regulatory authorities increasingly advocate for the reduction of animal use and the implementation of New Alternative Methods (NAMs), including microphysiological systems (MPS), to enhance the prediction of human-relevant liver toxicity (Shrimali et al., 2025).

In this context, we developed an innovative *in vitro* rat liver model designed to bridge the gap between cell-based assays and whole-animal studies while accounting for inter-animal variability. This model integrates multiple liver cell populations and leverages a recently established technology to generate individual-centric spheroids derived from a simple, minimally invasive blood sampling procedure (Cherradi et al., 2023; Roux et al., 2024a; b). By enabling the generation of personalized rat liver spheroids, our approach allows, for the first time, the parallel evaluation of a drug’s DILI potential both *in vitro* and *in vivo* within the same animal. To validate the model, we selected two mechanistically distinct and clinically relevant hepatotoxicants: Diclofenac, a nonsteroidal anti-inflammatory drug associated with idiosyncratic, metabolite- and immune-mediated liver injury (Boelsterli, 2003), and Bosentan, which induces dose-dependent cholestatic injury through bile salt export pump (BSEP) inhibition (Fattinger et al., 2001). The use of these compounds enables comprehensive assessment of the model’s capacity to detect both unpredictable and mechanistically defined forms of DILI.

## Materials and methods

### Animals

All procedures to be followed for conduct of this study were in accordance with the standard operating procedures followed at PRADO and all the *in vivo* procedures were as per the guidelines set by the Committee for Control and Supervision of Experiments on Animals (1723/PO/RcBiBt/S/13/CCSEA), Department of Animal Husbandry and Dairying, Ministry of Fisheries, Government of India for conducting experiments on small laboratory animals as published in ‘The Gazette of India’, 15 December 1998. Prior approval of the Institutional Animal Ethics Committee (IAEC) was taken (IAEC-25-056). The study was conducted in Sprague Dawley rats of both sexes. A total of 30 animals (15 males and 15 females), 6–8 weeks of age and weighing 130–240 g at initiation of dosing, were obtained from an accredited Animal Research Facility. Females were nulliparous and non-pregnant. Animals were housed under controlled environmental conditions (22 °C ± 3 °C; 30%–70% humidity; 12 h light/12 h dark cycle) in polysulfone cages with up to three animals per sex per cage. Standard rodent diet and reverse-osmosis water were provided *ad libitum*. Animals were acclimatized for at least 5 days prior to randomization into treatment groups based on body weight. The experimental rats used in this study were procured from the National Institute of Biosciences, Pune, Maharashtra, India. Table 1 summarizes the dose groups, administered drug concentrations, number of animals per group, and sex distribution.

**TABLE 1:** Overview of dose groups, drug concentrations, animal numbers, and sex distribution.

​	​	*In vivo*		*In vitro*	
Animal ID	Sex	Group	Dose	Group	Dose range
112,001	Male	Control (CMC)	0.2%	The *in vivo* control group was not used *in vitro*; each rat serves as its own control, with DMSO-treated spheroids representing baseline conditions	​
112,002	Male	Control (CMC)	0.2%	​	​
112,003	Male	Control (CMC)	0.2%	​	​
112,004	Male	Control (CMC)	0.2%	​	​
112,005	Male	Control (CMC)	0.2%	​	​
112,006	Female	Control (CMC)	0.2%	​	​
112,007	Female	Control (CMC)	0.2%	​	​
112,008	Female	Control (CMC)	0.2%	​	​
112,009	Female	Control (CMC)	0.2%	​	​
112,010	Female	Control (CMC)	0.2%	​	​
112,021	Male	Diclofenac	10 mg/kg	Diclofenac	0.01× to 100× Cmax
112,022	Male	Diclofenac	10 mg/kg	Diclofenac	0.01× to 100× Cmax
112,023	Male	Diclofenac	10 mg/kg	Diclofenac	0.01× to 100× Cmax
112,024	Male	Diclofenac	10 mg/kg	Diclofenac	0.01× to 100× Cmax
112,025	Male	Diclofenac	10 mg/kg	Diclofenac	0.01× to 100× Cmax
112,026	Female	Diclofenac	10 mg/kg	Diclofenac	0.01× to 100× Cmax
112,027	Female	Diclofenac	10 mg/kg	Diclofenac	0.01× to 100× Cmax
112,028	Female	Diclofenac	10 mg/kg	Diclofenac	0.01× to 100× Cmax
112,029	Female	Diclofenac	10 mg/kg	Diclofenac	0.01× to 100× Cmax
112,030	Female	Diclofenac	10 mg/kg	Diclofenac	0.01× to 100× Cmax
112,031	Male	Bosentan	60 mg/kg	Bosentan	0.01× to 100× Cmax
112,032	Male	Bosentan	60 mg/kg	Bosentan	0.01× to 100× Cmax
112,033	Male	Bosentan	60 mg/kg	Bosentan	0.01× to 100× Cmax
112,034	Male	Bosentan	60 mg/kg	Bosentan	0.01× to 100× Cmax
112,035	Male	Bosentan	60 mg/kg	Bosentan	0.01× to 100× Cmax
112,036	Female	Bosentan	60 mg/kg	Bosentan	0.01× to 100× Cmax
112,037	Female	Bosentan	60 mg/kg	Bosentan	0.01× to 100× Cmax
112,038	Female	Bosentan	60 mg/kg	Bosentan	0.01× to 100× Cmax
112,039	Female	Bosentan	60 mg/kg	Bosentan	0.01× to 100× Cmax
112,040	Female	Bosentan	60 mg/kg	Bosentan	0.01× to 100× Cmax

Diclofenac Cmax = 4.29 µM (PMID: 28781777).

Bosentan Cmax = 0.19 µM (PMID: 28156176).

The *in vivo* part was conducted in compliance with good laboratory practice (GLP) norms and following OECD GL 407 ‘Repeated Dose 28-day Oral Toxicity Study in Rodents’ 25 June 2025.

## Blood sampling and serum preparation

Blood was collected from rats prior to any drug administration, and serum was isolated and stored until use. The blood collected was left to clot for 30 min at 37 °C and centrifuged at 2,000 G at +4 °C for 10 min. The serum was collected, filtered through 0.45 µm, and then stored at −80 °C until used for *in vitro* experiment. The serum therefore did not contain diclofenac, bosentan, or their metabolites. Individual sera were used to educate rat liver spheroids *in vitro* before compound exposure, with the aim of conditioning the cells with animal-specific baseline systemic factors while keeping drug exposure strictly controlled during the *in vitro* phase.

### Drug administration

Diclofenac was administered orally for 28 consecutive days with 10 mg/kg/day, a dose previously shown to induce measurable hepatic effects in rats, including elevated liver enzymes and histopathological changes consistent with diclofenac-induced liver injury (Kadhim et al., 2022). Bosentan was administered orally for 28 consecutive days with 60 mg/kg/day, a dose selected to achieve sufficient hepatic exposure and stress in rats, accounting for species differences in metabolism and clearance (Fattinger et al., 2001). These dosing regimens are within ranges commonly used in preclinical studies to model drug-induced liver injury, acknowledging that rodent doses often exceed human therapeutic equivalents to elicit organ stress within a practical experimental timeframe (Pan et al., 2019). Animals in control group were administered with vehicle (0.2% carboxymethylcellulose (CMC)). CMC provides a stable suspension for poorly water-soluble compounds and has minimal pharmacological activity, making it suitable for repeated dosing regimens in preclinical studies. Oral gavages (16–18 gauge) needle fitted with a graduated syringe were used. The dose volume was maintained at 10 mL/kg body weight and actual volume to be administered was calculated based on the recent body weights of each animal.

Body weights of all animals were recorded once in a week till terminal sacrifice and body weight gains were calculated (Hoffman et al., 2002).

### Clinical chemistry

After completion of 28 days of dose administration, fasting blood samples were collected from all animals in vials containing heparin (250 IU/mL) as an anticoagulant for plasma preparation. Plasma samples were then processed using Erba EM 200 Clinical Chemistry Analyzer (Transasia Bio-Medicals Ltd., Mumbai, IN) to quantify ALT, AST, ALP, albumin, and bilirubin. In the present study, AST, ALT, ALP, albumin, and bilirubin were not measured or interpreted as mechanistic indicators of hepatotoxicity. Instead, these biomarkers were used strictly as clinical reference anchors to stratify compounds according to their documented *in vivo* severity. This approach allowed us to categorize and benchmark *in vitro* responses against recognized clinical injury patterns, providing a translational framework for evaluating compound-specific cytotoxicity without implying mechanistic insight from these serum parameters.

## Liver weights

During necropsy, liver from all animals were collected and weighed.

## Generation of rat liver spheroids

Serum samples collected from individual rats were used to condition rat-derived hepatocyte, hepatic stellate cell, and macrophage lines during spheroid formation. This serum-mediated education aims to induce phenotypic and metabolic changes in the rat cells, thereby enabling the resulting rat liver spheroids to closely replicate the metabolic state of the respective donor animal. Each rat liver spheroid contains a mixed population of rat cells. Rat liver spheroids were generated in 384-well ultra-low attachment plates. Each well was seeded with 4,500 hepatocytes (Clone 9), 500 fibroblasts (BRL 3A), and 50 macrophages/mL (NR8383). All cell lines are from ATCC. Cells were allowed to assemble into spheroids over 3 days. To illustrate the effect of hepatocyte number on spheroid size, Supplementary Figure S2 presents representative images of spheroids generated with 4,500 and 9,000 hepatocytes, allowing a comparison of spheroid dimensions 3 days post-seeding.

Following assembly, compounds were applied for exactly 3 days, with the medium refreshed once at the start of treatment and maintained without further changes throughout the treatment period. Rat serum was included (50% v/v) in the hSELS medium (PredictCan Biotechnologies, Grabels, France) for the entire duration of the *in vitro* experiment to support physiological relevance. The rat liver spheroids used in this study were generated from well-established rat cell lines that have been previously characterized and used for studies of liver biology, toxicology, and cellular responses (Lane et al., 1998; Sahu et al., 2008; Zhang et al., 2017).

A pooled serum experiment was performed by combining 30 µL of serum from each rat within the group to generate a working pooled serum sample. This pooled serum was then used at 50% (v/v), diluted in hSELS medium, to educate the rat spheroids. The experimental procedure was otherwise identical to that performed using individualized serum from each rat.

The term “serum-educated” is used deliberately and consistently with the cell-educating technology originally developed and validated in human models (Cherradi et al., 2023; Roux et al., 2024a; b). In this framework, exposure to serum is not a simple treatment or priming step, but a conditioning phase during which cells undergo measurable and stable phenotypic adaptations. This approach ensures that spheroids acquire individualized characteristics reflective of the donor serum prior to subsequent drug exposure.

## Treatment of rat liver spheroids

Drug dosing was performed by adding the drugs directly to the cell culture medium containing rat serum, where the serum originated from blood collected before the rats were treated and therefore contained no drugs or metabolites. A standard 3-day treatment protocol was applied to rat liver spheroids, with each concentration tested in technical triplicate to ensure reproducibility. Rat liver spheroids were exposed to diclofenac or bosentan across a ten-point concentration range with DMSO used as control. The final DMSO concentration was maintained at <2%, even at the highest drug dose. Diclofenac and bosentan were tested across a range of concentrations to capture sub-therapeutic, therapeutic, and supra-therapeutic exposures. Diclofenac concentrations were 0.0429 µM, 0.429 µM, 2.145 µM, 4.29 µM, 8.58 µM, 21.45 µM, 42.9 µM, 214.5 µM, and 429 μM, corresponding to 0.01x, 0.1x, 0.5x, 1x, 2x, 5x, 10x, 50x, and 100x C_max_, respectively (C_max_ = 4.29 µM). Bosentan concentrations were 0.0019 µM, 0.019 µM, 0.095 µM, 0.19 µM, 0.38 µM, 0.95 µM, 1.9 µM, 9.5 µM, and 19 μM, corresponding to 0.01x, 0.1x, 0.5x, 1x, 2x, 5x, 10x, 50x, and 100x C_max_, respectively (C_max_ = 0.19 µM). Concentration selection was guided by previously published pharmacokinetic studies (Hanna et al., 2016; Yuan et al., 2017) to ensure relevance to systemic exposure. Although direct translation to hepatic exposure in rats is limited by interspecies differences in metabolism, this concentration range allows assessment of effects at physiologically relevant exposures and at higher levels that may reveal potential toxicity mechanisms *in vitro*.

A key component of our spheroid culture protocol is the serum-education phase, implemented over the initial 3 days of spheroid formation. This period is sufficient for cells to undergo stable, serum-driven phenotypic adaptation. (Cherradi et al., 2023; Roux et al., 2024a; b). Variability observed in spheroids after drug exposure is therefore interpreted as the consequence of pre-established, serum-mediated conditioning, rather than stochastic or speculative differences in the cell population. In the present study, we applied this serum-education strategy to rat liver cell line-based spheroids to prepare the system for the assessment of drug-induced liver injury.

### Cell viability

Cell viability is a widely accepted indicator of drug-induced liver toxicity, as it reflects the overall health and metabolic competence of hepatocytes within the spheroids. We assessed viability using the CellTiter-Glo assay (Promega), which quantifies cellular ATP levels, a direct measure of metabolically active, viable cells. This assay is compatible with 3D liver spheroids and has been successfully used in previous studies to assess viability and hepatotoxic responses, providing reliable and reproducible measurements while better preserving liver-like physiology compared with traditional 2D cultures (Bell et al., 2018). By applying CellTiter-Glo across multiple drug concentrations and time points, we can establish dose-response relationships and identify concentration thresholds at which drugs induce hepatocellular injury, providing valuable insights into their potential hepatotoxicity.

### Data analysis

For *in vivo* analysis, body weight, liver weight, and biochemical marker results from vehicle and treated groups are presented as box plots to display the median, interquartile range, and potential outliers. Statistical comparisons between groups were performed using the Mann–Whitney test, a non-parametric method suitable for small sample sizes and non-normally distributed data typical of rat studies.

For *in vitro* analysis, data were collected and normalized to control DMSO condition. These datasets were then input into PredictCan-MIND to generate non-linear curve fitting and determine DILI severity of the tested compound.

Associations between *in vitro* and *in vivo* data were evaluated using simple linear regression to visualize the relationship, and the coefficient of determination (R^2^) was reported. Statistical significance of the correlation was assessed using F-test.

To evaluate the linear relationship between *in vitro* severity and *in vivo* biochemical markers, the *in vitro* datasets were processed using PredictCan-MIND, a proprietary platform from PredictCan (Microphysiological Intelligence for Networked Diversity). PredictCan-MIND generated a predicted DILI severity score, based on the percentage of dead cells, for each individual rat spheroid, enabling a direct comparison between cellular responses *in vitro* and *in vivo* liver injury outcomes.

## Results

### Diclofenac-induced DILI escapes detection *in vivo* but is captured by metabolically educated rat *in vitro* models

**FIGURE 1:**
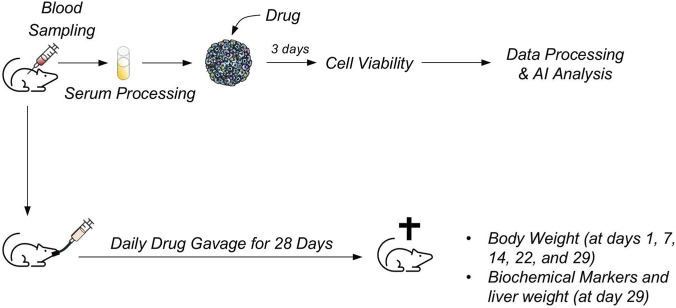
Workflow of the rat *in vitro*-*in vivo* experiment using serum-educated spheroids. The schematic illustrates the integrated *in vitro* and *in vivo* workflow. Individual rat serum was collected prior to any drug treatment and used to generate serum-educated spheroids, ensuring that the cellular conditioning reflects each animal’s baseline physiology rather than drug-induced changes. These spheroids were then exposed to test compounds *in vitro* to assess early signals of toxicity, such as reductions in ATP indicative of compromised cell viability, before corresponding *in vivo* endpoints were measured. The goal of this approach is to capture individualized, pre-existing susceptibility to drug-induced liver injury in a controlled cellular environment, while linking these *in vitro* responses to physiologically integrated *in vivo* outcomes, thereby improving predictive toxicology and mechanistic understanding.

**FIGURE 2:**
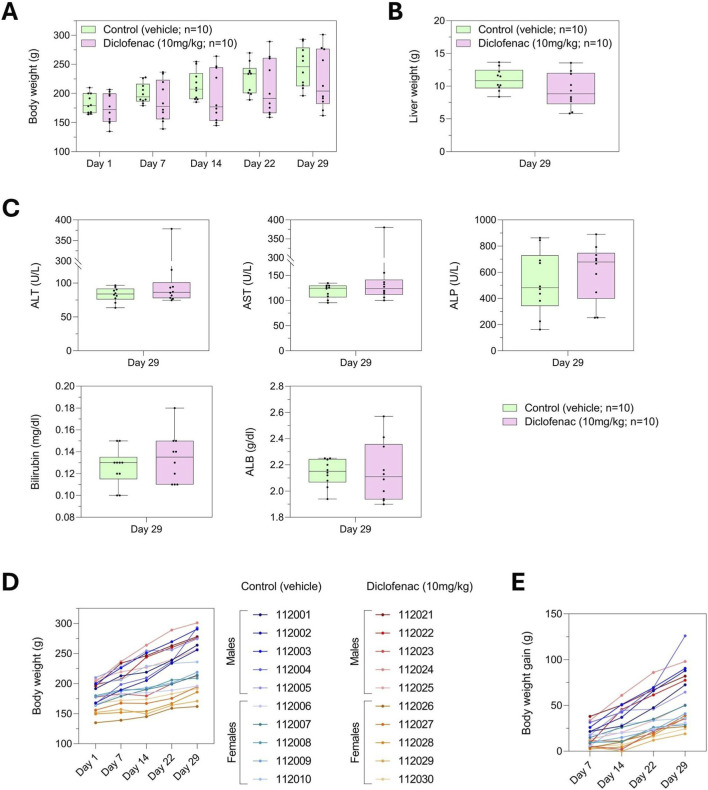
Group-based assessment in diclofenac-treated rats failed to detect overt hepatotoxicity, suggesting that subtle or individual-specific liver effects may be masked in average measurements. **(A)** Body weight of the rats was recorded over the entire duration of the study. Results are presented as box plots, showing median, interquartile range, and overall variability. **(B)** Liver weight was measured at necropsy at the end of the study, and the data are presented as a box plot to show distribution and variability among animals. **(C)** Biochemical markers, including ALT, AST, ALP, albumin, and bilirubin, were measured at study termination on day 29. Results are presented as box plots. **(D)** Body weight of each rat was monitored throughout the study, and the graph shows individual trajectories over time. **(E)** Body weight gain profiles for each rat across the entire study period are shown.

Figure 1 illustrates the overall workflow of our study, integrating both the *in vivo* and *in vitro* components. The schematic depicts the generation of individualized rat liver spheroids using the cell educating technology with rat serum, treatment *in vitro*, and the parallel *in vivo* evaluation of drug-induced liver injury in the corresponding animals. In the *in vivo* experiment, at group-level analysis, diclofenac treatment led to a reduction in median body weight and liver weight, although these changes were not statistically significant (Figures 2A,B). Classical serum markers of liver injury, including AST, ALT, and bilirubin, showed no detectable elevation, indicating that overt hepatocellular damage was absent under these experimental conditions. We observed that albumin exhibited a slight decreasing trend, while ALP showed a minor increase, but neither reached statistical significance (Figure 2C). Importantly, when body weight was analyzed at the individual animal level, some diclofenac-treated rats displayed more flattened growth curves compared with controls, despite similar food consumption across all groups (Supplementary Figure S1), suggesting a potential inter-individual susceptibility to diclofenac effects rather than a palatability-related issue (Figure 2D).

**FIGURE 3:**
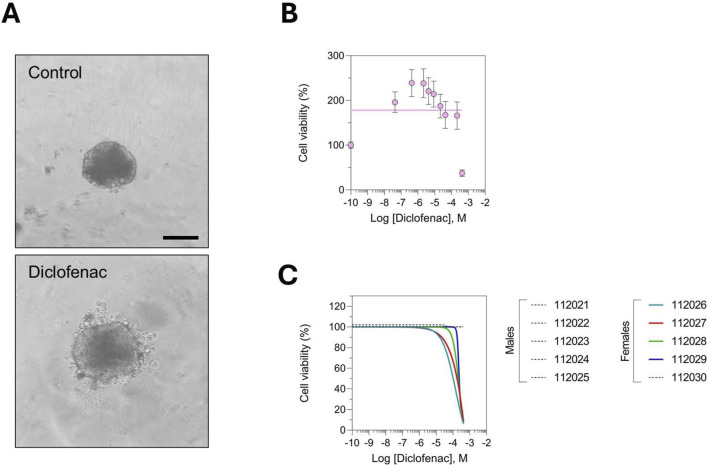
*In vitro* rat liver spheroids demonstrated that diclofenac induced DILI in some animals highlighting inter-individual susceptibility. **(A)** Representative images of rat liver spheroids in control (DMSO) and upon diclofenac treatment showing a loss of integrity of the structure of the spheroid. Scale bar represents 100 µm. **(B)** Diclofenac-induced liver injury was not detected in group-level analyses, likely reflecting a dilution effect from unaffected animals. Results are displayed as nonlinear curve fits. Each data point represents the mean ± s. e.m. of a technical triplicate. **(C)** Nonlinear curve fits for individual rats show that a subset developed diclofenac-mediated DILI highlighting inter-animal variability in susceptibility. The study included nine diclofenac doses and a control DMSO, with each condition measured in technical triplicate.

We next evaluated our novel *in vitro* rat models, generated using blood serum from the same animals analyzed *in vivo*. Rat liver spheroids were treated with increasing concentrations of diclofenac for 3 days, and cell viability was assessed. Figure 3A shows a spheroid with peripheral disintegration and cells detaching from the main body, indicating a loss of structural integrity following diclofenac treatment. The spheroids were composed of hepatocytes, stellate cells, and macrophages in a 90:10:1 ratio, with hepatocytes forming the majority of the cellular mass. While we cannot confirm the identity of the disintegrating cells due to the lack of cell-type-specific imaging, it is reasonable to infer that hepatocyte injury predominates. These morphological changes reflect overall spheroid integrity and were assessed as part of the global viability readout, which was the primary endpoint of the study. Data were analyzed using non-linear curve fitting to accurately capture dose-response relationships. Consistent with the *in vivo* findings, group-level analysis did not reveal significant diclofenac-induced DILI (Figure 3B). Similarly, pooled serum analysis, in which rat sera were pooled prior to the education of cell line-based spheroids, produced results consistent with those observed in the group-level analyses (Supplementary Figure S3A). However, when DILI risk was analyzed at the individual animal level, 4 out of 10 rats in the treated group exhibited reduced cell viability, confirming their susceptibility to diclofenac-mediated hepatotoxicity. Interestingly, all 4 susceptible rats were females, while no males showed liver toxicity, highlighting a clear sex-related difference (Figure 3C).

**FIGURE 4:**
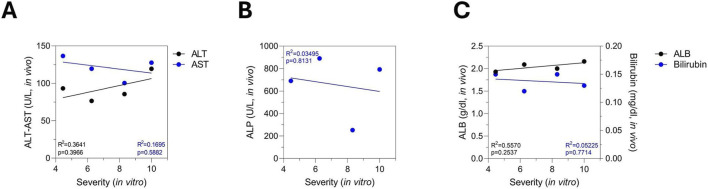
Linear regression analysis indicates a lack of correlation between *in vivo* biochemical markers and *in vitro* toxicity endpoints suggesting that standard biomarkers may not predict individual susceptibility. **(A)** *In vivo* ALT and AST, **(B)** ALP, **(C)** albumin and bilirubin, were quantified for each rat, while *in vitro* toxicity severity was assessed using PredictCan-MIND based on data from the same diclofenac-treated animals generating paired datasets for simple linear regression analysis. Only rats with detectable *in vitro* toxicity were included in the linear regression to prevent dilution by severity metric of zero values (absence of DILI), which represent the categorical absence of injury rather than continuous measurements thereby avoiding artificial compression of the data range and obscuring the correlation. Shown are the coefficient of determination (*R* ^2^) and F-test p-value.

We analyzed the correlation between *in vitro* severity, determined by PredictCan-MIND, and the *in vivo* biochemical makers. We observed no significant correlation between *in vitro* DILI severity and classical liver injury markers, including AST, ALP, albumin, and bilirubin (Figures 4A–C). A slight trend of correlation was noted for ALT (R^2^ = 0.3641, p = 0.3966), but this did not reach statistical significance (Figure 4A). Importantly, this analysis included only 4 rats, as these were the only animals exhibiting cell death *in vitro* (Figure 3B).

### *In vitro* rat model reveals bosentan-induced cholestatic injury in individual rats consistent with *in vivo* ALP measurements

**FIGURE 5:**
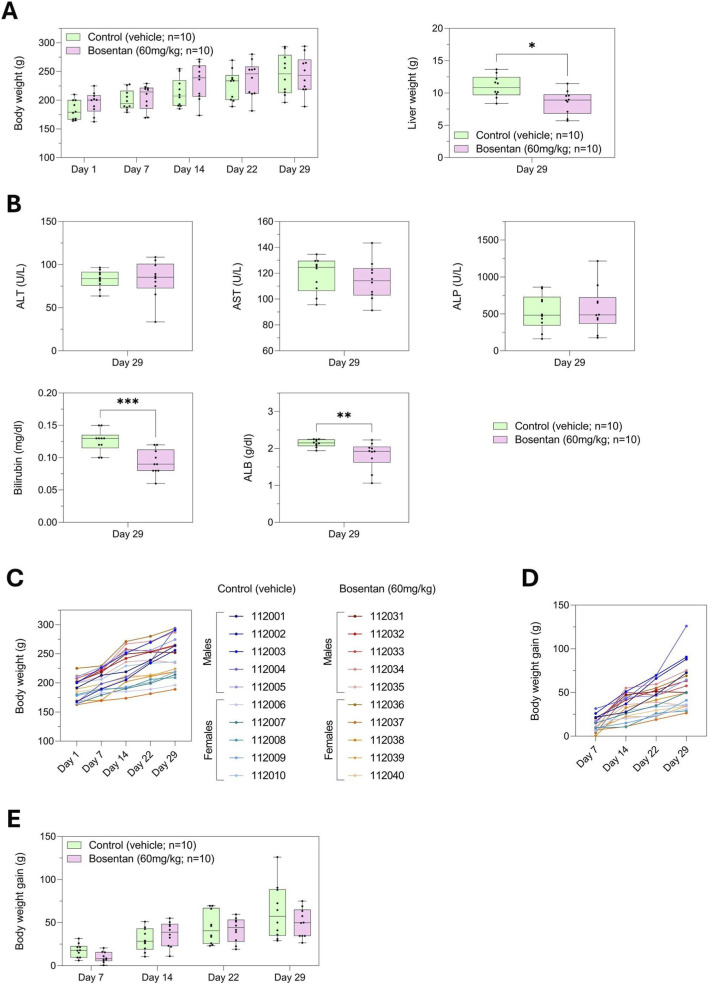
Analysis at the group level showed changes in liver weight and certain biochemical parameters in rats treated with bosentan, highlighting possible hepatotoxic effects. **(A)** Body weights were measured throughout the study. Liver weights were measured at termination of the study. Results are shown as box plots. *p < 0.05, Mann-Whitney test. **(B)** Biochemical markers including ALT, AST, ALP, albumin, and bilirubin. Results are shown as box plots. **p < 0.01, ***p < 0.001 Mann-Whitney test. **(C)** Longitudinal body weight and **(D)** body weight gain profiles for each rat across the entire study period are shown. **(E)** Group analysis of body weight gain across the study period. Results are shown as box plots.

We evaluated the performance of our *in vitro* rat spheroid model using bosentan, a compound that carries a higher relative risk of DILI compared with diclofenac. As previously performed with diclofenac, we conducted a prospective study with bosentan, in which blood serum was collected from rats prior to treatment with 60 mg/kg orally. Serum-educated rat liver spheroids were then generated for each animal, and a dose-response analysis was performed to assess DILI risk. *In vivo*, bosentan treatment did not affect rat body weight (Figure 5A). Liver weight was statistically reduced in the bosentan-treated group compared with controls, potentially indicating a liver effect, though histopathology would be required to confirm injury (Figure 5A). Further group-level analysis revealed that albumin levels were significantly decreased in the bosentan-treated group compared with controls. No significant changes were observed in ALT, AST, or ALP, indicating the absence of a consistent group-level biochemical signature of liver injury. Surprisingly, a reduction in bilirubin levels was also observed in bosentan-treated rats, contrary to the typical pattern expected for cholestatic injury (Figure 5B). The unexpected decrease in bilirubin levels, together with the group-level reduction in albumin, suggests that bosentan-induced liver effects may not manifest uniformly across animals. However, when body weight trajectories were examined individually, several bosentan-treated rats displayed flattened growth curves compared with controls (Figure 5C), indicating a trend toward reduced body weight gain in the treated group (Figure 5D), although this difference did not reach statistical significance (Figure 5E).

**FIGURE 6:**
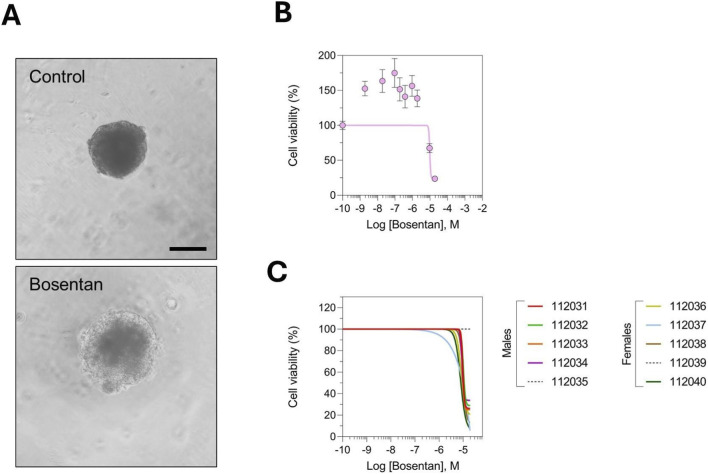
*In vitro* rat liver spheroid experiments showed that bosentan induced DILI in only some animals underscoring the role of inter-individual variability in drug response. **(A)** Representative images of rat liver spheroids in control (DMSO) and upon bosentan treatment showing an alteration of the structure of the spheroid. Scale bar represents 100 µm. **(B)** Group-level analysis shows that bosentan treatment caused DILI indicating that the dilution effect was mitigated by the high incidence of liver toxicity. Results are displayed as nonlinear curve fits. Each data point corresponds to the mean ± s. e.m. of a technical triplicate. **(C)** Examination of data at the individual animal level corroborated a high incidence of DILI induced by bosentan. Shown are nonlinear curve fits for individual rats. The study included nine bosentan doses and a control DMSO, with each condition measured in technical triplicate.

We next performed *in vitro* analyses using liver spheroids generated from the same rats included in the *in vivo* study. Figure 6A shows representative images of structural alteration of rat liver spheroids upon 3 days of treatment with bosentan. Group-level analysis revealed a significant decrease in cell viability, confirming bosentan-mediated hepatotoxicity in our *in vitro* system (Figure 6B). Similar results were obtained in the pooled serum experiment (Supplementary Figure S3B). When the data were examined at the individual level, liver spheroids from 8 out of 10 rats exhibited clear signs of cell death following bosentan exposure, further validating the compound’s toxic potential (Figure 6C). In this case, and contrary to the previous diclofenac study, the observation of a group-level effect is expected, as the majority of rats showed a consistent *in vitro* toxic response to bosentan, thereby amplifying the signal at the population level. Interestingly, no sex-related differences were observed in the *in vitro* response, which aligns with clinical observations showing that both men and women are at comparable risk of bosentan-induced DILI.

**FIGURE 7:**
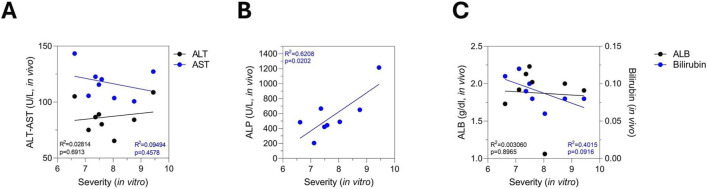
Simple linear regression shows a significant positive correlation between *in vivo* ALP levels and *in vitro* toxicity supporting that rat liver spheroids capture bosentan-induced cholestatic injury and can serve as a validated, non-lethal alternative to *in vivo* studies, using spheroids generated from the same animals’ serum. **(A)** *In vivo* ALT and AST, **(B)** ALP, **(C)** albumin and bilirubin, were quantified for each rat, while *in vitro* toxicity severity was assessed using PredictCan-MIND based on data from the same bosentan-treated animals generating paired datasets for simple linear regression analysis. Only rats with detectable *in vitro* toxicity were included in the linear regression. Shown are the coefficient of determination (*R* ^2^) and F-test p-value.

Since a larger proportion of rats exhibited liver toxicity *in vitro* following bosentan treatment, we performed a linear correlation analysis between *in vitro* DILI severity and *in vivo* biochemical markers, as the increased number of affected animals provided a sufficient number of paired values for meaningful analysis. As previously described, *in vitro* datasets were processed using PredictCan-MIND to determine individual DILI severity scores from the *in vitro* data. We observed no clear correlation between *in vitro* DILI severity and the *in vivo* biochemical markers ALT, AST, albumin, and bilirubin (Figures 7A,C). Interestingly, we observed a positive correlation between *in vitro* DILI severity and *in vivo* ALP levels, a well-established biochemical marker of cholestatic injury (Figure 7B). The correlation showed an R^2^ > 0.6 with statistical significance (p = 0.0202), indicating a strong linear relationship.

## Discussion

The present study aims to use a multicellular rat liver spheroid system obtained by using rat blood serum to educate co-culture of rat cell lines to assess hepatotoxic responses to selected compounds, focusing on integrated cellular viability outcomes, rather than to develop a new liver spheroid model. We evaluated rat *in vivo* and *in vitro* models for drug-induced liver injury using diclofenac and bosentan as representative hepatocellular and cholestatic compounds, respectively. Diclofenac at 10 mg/kg orally did not elicit measurable elevations in ALT, AST, ALP, or bilirubin in rats, consistent with the species’ relative resistance to idiosyncratic hepatotoxicity at moderate doses (Kishida et al., 2012; Tu et al., 2021). Nevertheless, our *in vitro* rat spheroid models revealed clear cell death in spheroids generated with blood serum from some animals. These findings are consistent with diclofenac-induced hepatotoxicity, as evidenced by reductions in ATP-based cell viability, without implying a specific underlying mechanism. We acknowledge that cell viability does not identify the intracellular pathways leading to injury. However, the primary objective of this study was to assess whether the multicellular rat liver spheroid system can discriminate compounds according to overall hepatotoxic liability. For this purpose, reduction in viability represents an integrative endpoint reflecting cumulative cellular stress, including mitochondrial dysfunction, oxidative stress, cholestatic injury, and inflammatory amplification. Viability was therefore intentionally selected as a global toxicity readout, consistent with many established *in vitro* DILI screening frameworks. These results suggest that our advanced biodiversity-capturing *in vitro* platform could further identify susceptible subpopulations highlighting the idiosyncratic nature of diclofenac-induced toxicity and improving translational relevance. In contrast, bosentan administered at 60 mg/kg via oral gavage in rats induced a predominantly cholestatic response, evidenced by ALP elevation without marked ALT/AST changes. The observed ALP increase without concomitant transaminase elevation highlights the early or subclinical cholestatic phase of injury, reflecting a mechanistically relevant response that is characteristic of transporter-mediated DILI (Sundaram and Bjornsson, 2017). This pattern aligns with bosentan’s BSEP inhibition-mediated mechanism and demonstrates that the rat model can reliably capture cholestatic DILI endpoints. Importantly, hepatocyte stress observed in our *in vitro* model correlated with the magnitude of ALP elevation *in vivo*, indicating that the *in vitro* platform can reproduce mechanistically relevant, cholestasis-related injury signals. Moreover, while conventional group-level *in vivo* analysis failed to detect clear treatment effects, notable inter-individual variability in drug response was observed among the animals. Our data suggest that diclofenac and bosentan do not induce overt DILI at the group level, highlighting the limited sensitivity of conventional *in vivo* biomarkers (Aubrecht et al., 2013; Chen Y. et al., 2022). Importantly, these results support the value of serum-educated *in vitro* rat models, which can capture individual metabolic variability and identify susceptible animals, offering a more sensitive and predictive approach for detecting early or idiosyncratic DILI signals. Together, these findings underscore that our rat *in vitro* assays are particularly useful for detecting intrinsic hepatocyte susceptibility, while rat *in vivo* models provide integrated physiological and cholestatic responses. Biodiversity-aware *in vitro* systems enhance predictive power by capturing population variability and idiosyncratic responses, and when their outputs correlate with *in vivo* biomarkers, they offer a mechanistically and translationally relevant bridge between preclinical testing and human DILI risk. This complementary strategy supports a more nuanced and predictive framework for DILI assessment, combining hepatocellular, cholestatic, and inter-individual susceptibility mechanisms.

Moreover, Leslie and colleagues demonstrated that bosentan inhibits rat NTCP more potently than human NTCP, providing a possible explanation for the generally low hepatotoxicity observed in rats (Leslie et al., 2007). Consistent with this, our *in vivo* data did not show significant changes in ALT and AST following bosentan treatment, although serum bile acids were not measured in our rats. Interestingly, *in vitro* rat liver spheroids generated using serum from the same animals exhibited reductions in cell viability, highlighting inter-individual variability and the ability of the spheroid system to capture cellular responses that may not be apparent in conventional *in vivo* biomarkers. These observations underscore the complementary nature of the *in vitro* rat liver spheroid platform for detecting hepatotoxic potential, with the ATP-based viability readout reflecting integrated cellular responses rather than specific intracellular mechanisms. We acknowledge that mechanistic data, such as transporter expression, metabolite formation, or adaptive responses, were not measured in this study. Accordingly, the spheroid data should be interpreted as providing early, cellular-level insights into compound-induced hepatotoxicity and inter-individual variability, complementary to *in vivo* studies, rather than as a mechanistic evaluation of cholestatic injury.

In the present study, we performed a correlation analysis exclusively on rats exhibiting liver injury, while rats with no detectable DILI (severity score set to 0 by PredictCan-MIND) were excluded. As these zeros do not represent true continuous measurements but rather the categorical absence of injury, including them would artificially compress the range of values, and consequently obscuring the strength of the relationship. Thus, by focusing on DILI-positive animals, the analysis isolates the true continuous variability in injury severity, enabling an accurate assessment of predictive performance. This approach ensures that the reported R^2^ reflects the biologically meaningful linear association between cellular responses and the degree of liver injury, rather than being diluted by the presence of structurally absent values.

In this study, only a small subset of rats exhibited measurable *in vitro* cell death. Given the limited number of paired observations between *in vitro* cytotoxicity and *in vivo* biochemical markers, the statistical power to detect a meaningful linear association was inherently low (Han et al., 2012; Stevens et al., 2018). Consequently, the lack of a significant correlation should not be interpreted as evidence of an absence of biological linkage, but rather as a limitation imposed by the small number of susceptible animals. This constraint underscores the importance of evaluating DILI at the individual level and highlights the need for larger datasets to robustly assess predictive relationships. The observed heterogeneity in cellular responses and variability in biochemical markers likely reflect subtle systemic or metabolic differences among individuals (Chen L. et al., 2022). Such variability suggests that group-averaged analyses may obscure individual susceptibilities, potentially masking treatment-related effects and complicating data interpretation. Therefore, assessing responses on an individual basis is essential to capture early or idiosyncratic manifestations of DILI and to identify animals at higher risk of hepatotoxicity. In this context, our serum-educated rat liver spheroid model, which incorporates the metabolic profile of each animal through its own serum, represents a valuable complementary approach. By recapitulating *in vivo* inter-individual variability, this platform enables a more personalized evaluation of hepatotoxic risk and facilitates the detection of early or subtle liver injury signals that may remain undetected in conventional group-based analyses.

Our *in vitro* rat cell line models, generated using the serum-based cell educating technology, accurately captured gender-related susceptibility to DILI, reflecting both animal- and human-relevant patterns. The models correctly identified that females are at higher risk of diclofenac-induced liver injury, whereas bosentan-induced DILI showed no significant difference between males and females. These observations are consistent with clinical data in humans, where diclofenac DILI occurs more frequently in women (Farkouh et al., 2021), while bosentan hepatotoxicity is largely gender-neutral (Gu et al., 2024). This alignment with human outcomes underscores the ability of our metabolically educated system to integrate individual metabolic profiles, including sex-dependent differences, and predict differential hepatotoxicity with high fidelity. In addition, for bosentan, we observed a strong linear relationship between the magnitude of *in vitro* cellular injury and the degree of cholestatic response measured *in vivo*, as reflected by ALP levels. This is particularly relevant given that bosentan is clinically recognized to induce cholestatic or mixed-pattern DILI through inhibition of BSEP and other hepatobiliary transporters (Fattinger et al., 2001; Mano et al., 2007; Markova et al., 2013). The concordance between *in vitro* cytotoxicity and *in vivo* cholestatic markers not only reinforces the translational robustness of our serum-educated rat liver spheroid model but also demonstrates its ability to recapitulate the mechanistic and phenotypic features of bosentan-induced liver injury observed in patients. Together, these findings highlight the robustness and predictive power of this platform for prospective identification of at-risk animals and support its potential to reduce reliance on conventional *in vivo* toxicity studies for detecting hepatotoxic effects.

In this study, we explicitly acknowledge the use of immortalized rat hepatocyte cell lines rather than primary hepatocytes. This choice is deliberate as it was made to evaluate whether our serum-mediated cell education approach could be applied to a rat system in order to capture inter-individual susceptibility to DILI, while ultimately contributing to the development of alternative *in vitro* strategies that may help reduce the need for animal experiments. Importantly, isolating primary hepatocytes would require sacrificing the animals, whereas blood sampling for serum collection is minimally invasive and does not require killing the animals, allowing us to model individual physiological conditions without additional animal loss. By combining standardized, well-characterized cells with individual rat serum in our proprietary cell-educating platform, we are able to capture subject-specific susceptibility to drug-induced liver injury while keeping the cellular background constant. This strategy, previously validated in human studies (Cherradi et al., 2023; Roux et al., 2024a; b), ensures that inter-individual variability in toxicity responses can be attributed to circulating serum factors rather than intrinsic differences in primary cells. The present work aims to assess whether this serum-driven personalization approach can be translated to a rat *in vitro*-*in vivo* framework for predictive toxicology. We acknowledge that rat cell lines are less metabolically competent than primary rat hepatocytes, and for this reason, we plan future comparative experiments evaluating primary hepatocytes alongside serum-educated cells to further assess the predictive value of the serum-education approach relative to intrinsic cellular variability.

The rat liver spheroid system used in this study is composed of hepatocytes, fibroblasts, and macrophages in a 90:10:1 ratio. Given that hepatocytes constitute the vast majority of the cellular mass, the ATP-based viability signal is predominantly driven by hepatocyte injury, while stellate cells and macrophages provide supportive and modulatory functions within the spheroid microenvironment. Consequently, reductions in global viability primarily reflect hepatocyte health, which is the principal target in most forms of drug-induced liver injury. We acknowledge that cell-type-specific analyses, such as targeted staining or flow cytometric separation, would offer deeper mechanistic resolution regarding which cells are affected. However, such approaches were beyond the scope of the present study, which was designed to assess translational toxicity ranking rather than mechanistic dissection. Future work incorporating cell-type-specific readouts could provide valuable insights into the contributions of non-parenchymal cells to DILI responses.

The use of rat cell lines such as rat hepatocytes, rat hepatic stellate cells, and rat macrophages, educated with serum from individual rats provides a unique window into individual metabolic susceptibility to DILI. The serum originates from the same animals used *in vivo*, the *in vitro* system is able to reveal hepatotoxic responses that may remain undetected in the whole animal. This discrepancy arises primarily from the presence of systemic compensatory mechanisms *in vivo* (Schug et al., 2013; Soldatow et al., 2013). Indeed, in a living rat, subtle hepatocyte injury can be masked by adaptive processes such as zonally localized liver compensation mechanism, where undamaged regions of the liver maintain functionality, upregulation of detoxifying enzymes that neutralize reactive metabolites, or enhanced excretion of bile acids and toxic intermediates, which prevents accumulation to levels that trigger detectable biomarker changes like plasma ALT, AST, or ALP (Russomanno et al., 2023). The primary objective of this study was to evaluate overall cellular viability as an integrative endpoint in multicellular rat liver spheroids exposed to compounds such as bosentan and diclofenac. Cell viability reflects cumulative cellular stress and cytotoxicity, providing a sensitive readout for early toxicity, but does not directly resolve specific intracellular mechanisms. The metabolically educated cell lines operate in a controlled, isolated environment, where systemic adaptations present *in vivo* are absent. Overall, these findings illustrate that even when derived from the same animals, *in vitro* models can reveal DILI risk that is obscured *in vivo* by systemic compensation, highlighting the power of metabolically educated cell lines to capture early, individualized hepatotoxic responses.

The stratification of hepatocyte spheroids into subpopulations based on ATP-based viability highlights inter-spheroid variability in response to diclofenac and bosentan. Some spheroids consistently exhibited greater reductions in viability, demonstrating that compound-specific hepatotoxicity can manifest differently even under identical experimental conditions. While the current study does not investigate the mechanistic basis of this variability, these subpopulations provide a valuable framework for exploring early and idiosyncratic hepatocyte responses in a controlled *in vitro* setting. Such stratification may inform future studies aimed at understanding factors that drive differential susceptibility and optimizing predictive *in vitro* models of hepatotoxicity.

Finally, the present findings highlight that the *in vitro* rat liver spheroid model captures inter-individual variability in hepatotoxic responses. Individual-derived spheroids exhibited variable reductions in viability, reflecting differences in susceptibility among animals, even when group-level *in vivo* biomarkers showed little or no change. Importantly, this observed variability underscores the potential of the *in vitro* system to explore early indicators of hepatotoxicity. While these findings do not provide full mechanistic insight or definitive predictive power, they support the use of such models as a complementary tool for investigating inter-individual susceptibility in a non-invasive, translational context. This advancement opens the possibility of preselecting or stratifying animals based on susceptibility prior to *in vivo* testing, thereby refining experimental design and minimizing unnecessary exposure to potentially hepatotoxic compounds. Given the strength of the correlation observed, the *in vitro* assay can be used to support a substantial reduction in animal numbers for validation studies, in alignment with the 3Rs principle and current regulatory perspectives on NAMs. Future studies involving multiple compounds and different organ systems as used routinely in repeated dose toxicity and different dosing paradigms will be essential to confirm the model’s generalizability and to advance its acceptance for regulatory applications in risk assessment and drug development.

## Data Availability

The datasets presented in this article are not readily available because The data generated in this study are not publicly available. Methods related to this study will be shared on reasonable request with permission of PredictCan Biotechnologies and PRADO. Requests to access the datasets should be directed to Hong Tuan DUONG, ht.duong@predictcan.com.
