# Supplementary figures and images for: A new in vitro rat liver platform capturing biological diversity for drug-induced liver injury assessment and reduction of animal use in drug discovery and development
Source: Front Toxicol. 2026 Apr 28;8:1784229. doi: 10.3389/ftox.2026.1784229 (PMC13160669; doi:10.3389/ftox.2026.1784229)

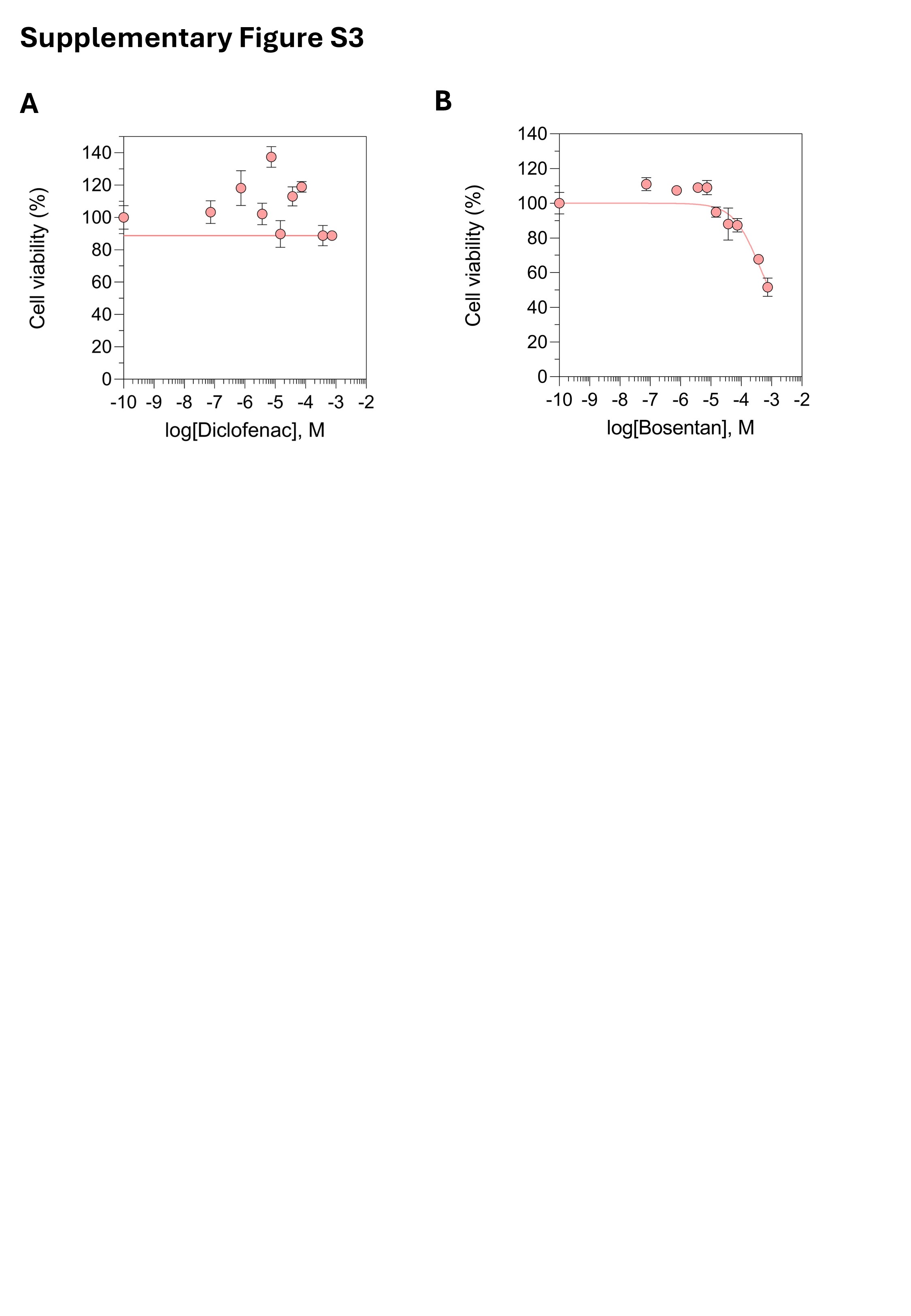

Supplement: Supplementary file 1 [file Image3.jpeg]

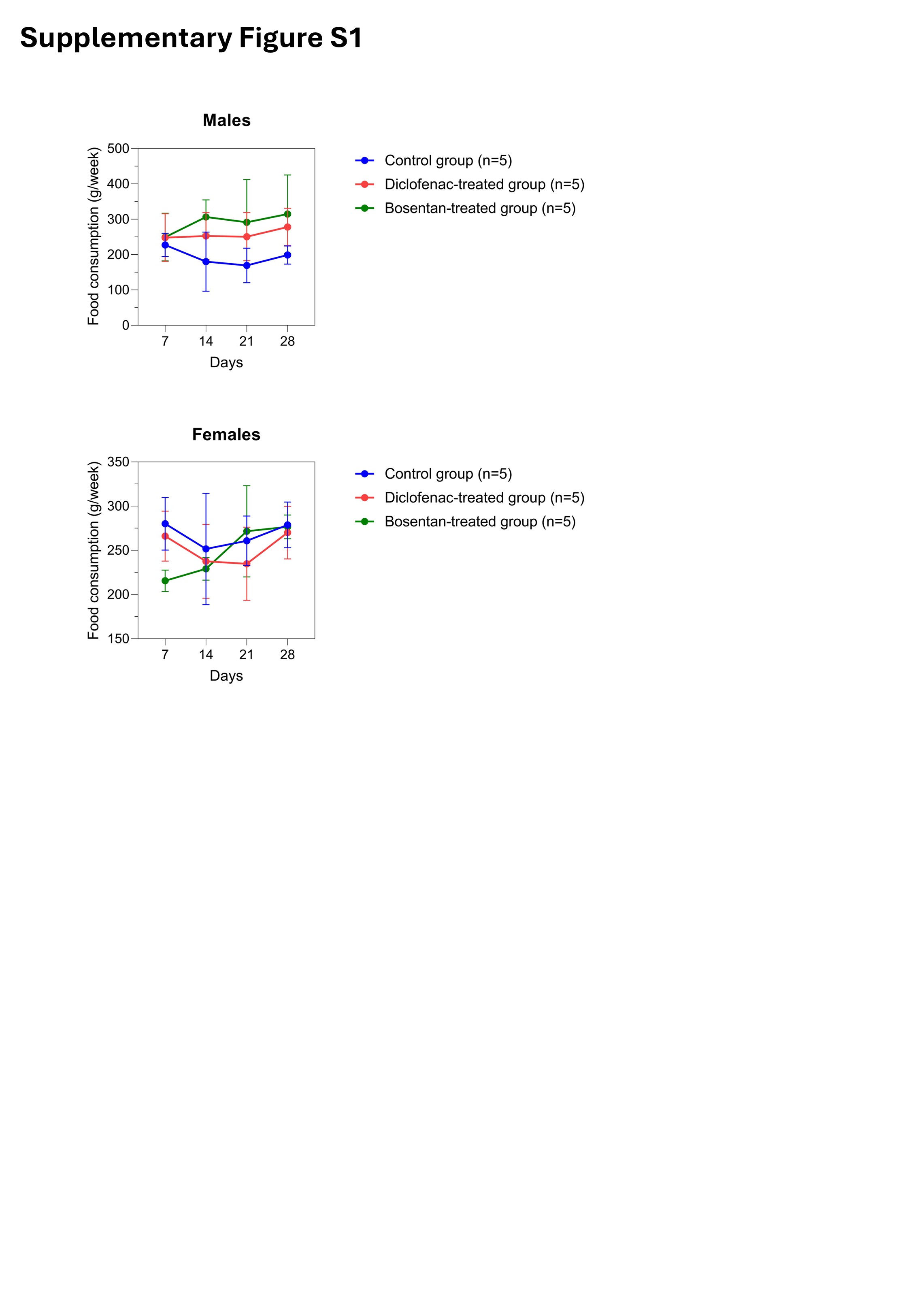

Supplement: Supplementary file 2 [file Image1.jpeg]

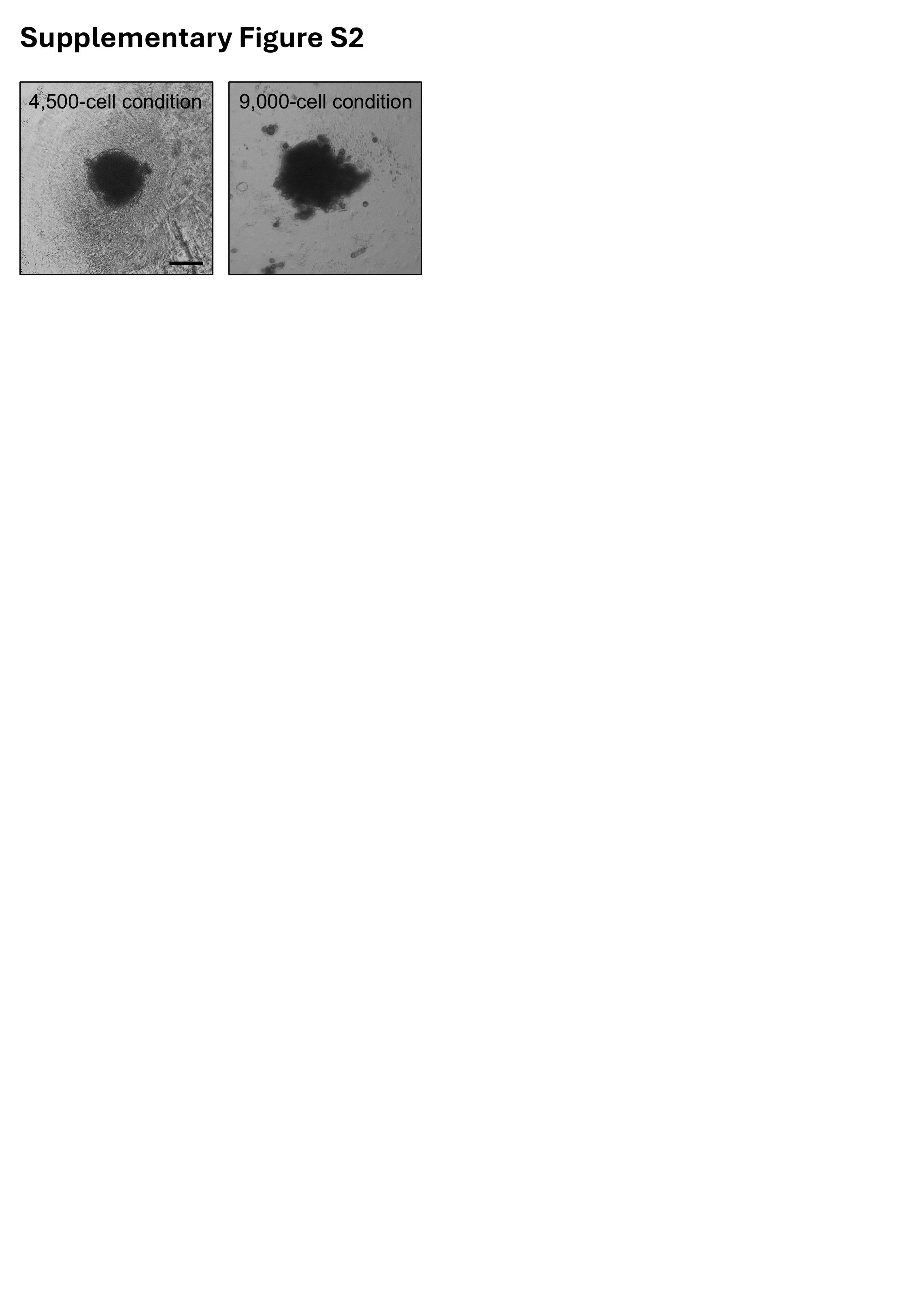

Supplement: Supplementary file 3 [file Image2.jpeg]
